# Development and psychometric evaluation of a learning needs assessment tool for healthcare professionals in palliative dementia care: A cross-sectional study

**DOI:** 10.1016/j.ijnsa.2025.100455

**Published:** 2025-11-14

**Authors:** Jesper M.A. Biesmans, Sascha R. Bolt, Sandra M.G. Zwakhalen, Daisy J.A. Janssen, Judith M.M. Meijers

**Affiliations:** a Department of Health Services Research, Care and Public Health Research Institute, Maastricht University, Maastricht, the Netherlands; b Living Lab for Aging and Long-term Care Limburg, Maastricht University, Maastricht, the Netherlands; c Tranzo, Tilburg School of Social and Behavioral Sciences, Tilburg University, Tilburg, the Netherlands; d Department of Family Medicine, Care and Public Health Research Institute, Faculty of Health, Medicine and Life Sciences, Maastricht University, Maastricht, the Netherlands; e Department of Expertise and Treatment, Proteion, Horn, the Netherlands; f Zuyderland Medical Center, Sittard-Geleen, the Netherlands; g Zuyd University of Applied Sciences, Heerlen, the Netherlands

**Keywords:** Dementia, Nursing, Palliative care, Psychometrics, Self-assessment

## Abstract

**Background:**

Palliative care aims to improve the quality of life of individuals with chronic, life-limiting diseases like dementia. Self-assessment by healthcare professionals of their learning needs helps to identify areas for improvement and enhance care provision. To support this, the Desired Dementia Care Towards End of Life (DEDICATED) questionnaire was developed; a self-assessment tool for measuring healthcare professionals’ skill development needs.

**Objective:**

To describe the development of the questionnaire and examine its psychometric properties.

**Design:**

Quantitative cross-sectional psychometric evaluation.

**Setting(s):**

Data was collected in nursing homes, hospital wards, and home care organizations providing palliative care to people with dementia in the Netherlands.

**Participants:**

The questionnaire was developed by healthcare professionals and researchers. Psychometric evaluation was then conducted with 332 Dutch healthcare professionals, divided over two samples.

**Methods:**

Scientific literature and expert input were used to develop the questionnaire, which then underwent feasibility testing and psychometric evaluation. Construct validity was assessed via exploratory and confirmatory factor analysis. Inter-item correlations were used to evaluate convergent validity, and item-factor correlations to assess discriminant validity. Reliability was tested using item-total correlations, Cronbach’s alpha, and McDonald’s omega. Ceiling effects and the tool’s ability to differentiate outcomes across healthcare professions were assessed with ANOVA. Pearson’s correlation was used to assess concurrent validity between the questionnaire and the End-of-Life Professional Caregiver Survey.

**Results:**

The 29-item questionnaire showed strong internal consistency, with a mean Cronbach’s alpha of .89 and McDonald’s omega of .90. Factor analysis identified five factors, explaining 71.68 % variance: (1) Familiarization with the person with dementia, (2) Timing for advance care planning, (3) Healthcare professional's role in advance care planning, (4) Interprofessional collaboration, and (5) Managing pain and responsive behavior. Statistically significant differences between nurses and nurse assistants suggest the questionnaire was able to differentiate outcomes across healthcare professions (mean difference = 6.15, 95 % CI: .15 to 12.2, *p* = .042). A moderate positive correlation was found between the questionnaire and End-of-Life Professional Caregiver Survey (*r* = .33, 95 % CI: .13 to .50, *p* = .002)

**Conclusion:**

The DEDICATED questionnaire shows promising psychometric properties and could support the needs of healthcare professionals in providing palliative care for people with dementia.

**Registration:**

Not Registered.

What is already known:

• Self-assessment of healthcare Professional's skills and competencies is a prerequisite for improving palliative care provision and can lead to learning goals and subsequent improvement of quality of care.
• However, current self-assessment questionnaires mostly focus on general knowledge of palliative dementia care and do not capture healthcare Professional's self-perceived skills and competencies related to the full care trajectory of palliative care for people with dementia.

What this paper adds:

• A novel self-assessment questionnaire based on theoretical research in palliative dementia care, focusing on identifying skills and competencies needed to provide person-centered palliative care throughout the palliative care trajectory. Development followed extensive psychometric evaluation to ensure healthcare professionals can gain insight into their needs and learning goals.
• Information into the experienced needs and learning goals (alongside the psychometric evaluation) of healthcare professionals working in the field of palliative care for people with dementia.
• This study revealed promising psychometric properties of the questionnaire and highlights its applicability for a broad range of healthcare professionals working in palliative care for people with dementia.

## 1 Background

Healthcare professionals work closely with people with dementia and their family caregivers to provide person-centered palliative care using a holistic approach (Brinkman-Stoppelenburg et al., 2014; Faull, 2024; Fryer et al., 2016; World Health Organization, 2025). However, complex palliative care needs or healthcare professionals’ limited knowledge about the disease can lead to challenges in the provision of care (Martin et al., 2020; Surr et al., 2017). They may also lack certain skills, such as those related to advance care planning conversations, pain assessments, or supporting people with dementia and their families throughout the care trajectory (Huijten et al., 2023; Khemai et al., 2020; Bolt et al., 2019a). Therefore, it is crucial that healthcare professionals receive adequate training and information to enhance their competencies and improve palliative care provision (Surr et al., 2017). An additional aspect for enhancing the quality of palliative care is healthcare professionals’ self-assessment and critical evaluation of their own needs and learning goals for competency improvement (Newble, 1992; Slåtten et al., 2014). These needs can be explored using self-assessment questionnaires, which can increase healthcare professionals’ awareness of their knowledge gaps and learning goals, and thereby stimulate workplace learning and quality improvement in palliative care.

However, existing self-assessment questionnaires regarding palliative care (Karacsony et al., 2018; Lazenby et al., 2012; Long et al., 2012; Sawatzky et al., 2021; Slåtten et al., 2014) primarily focus on evaluating general knowledge of the subject, rather than assessing the competencies and skills required for providing care for people with dementia over the course of the care trajectory. Moreover, they are not available in Dutch. This highlights the need for a comprehensive self-assessment questionnaire, focusing on skills and competencies relevant to the various domains of palliative dementia care (Biesmans et al., 2025). This paper describes the development and assesses the psychometric properties of a new self-assessment tool, called the Desired Dementia Care Towards End of Life (DEDICATED) questionnaire. Additionally, this study explores its potential usefulness for various healthcare professionals in the field of palliative care for people with dementia.

### 1.1 The DEDICATED project

The psychometric evaluation of the DEDICATED questionnaire is embedded within the broader DEDICATED research project. The DEDICATED project aims to enhance the quality of palliative care for people with dementia, by strengthening healthcare professionals’ knowledge and skills (Biesmans et al., 2024, 2025). The project provides a set of practical tools and a two-session training. Healthcare professionals who completed the DEDICATED training program become DEDICATED ambassadors, and through a train-the-trainer program, they can further qualify as trainers, thereby facilitating broader dissemination and sustainable implementation of the approach (Biesmans et al., 2024). Within the DEDICATED project, the DEDICATED questionnaire is used as a needs assessment for DEDICATED ambassadors and their direct care team members, to provide directions for which DEDICATED tools to use to improve the quality of care.

Prior to the development of the DEDICATED approach, several qualitative and quantitative studies were conducted to formulate key themes for high-quality palliative care for people with dementia. These studies investigated the perspectives of healthcare professionals, people with dementia, and their family caregivers, and served as the theoretical basis for the development of the DEDICATED approach (Bolt et al., 2019a; 2019b; Bolt et al., 2020, 2022; Khemai et al., 2020, 2022). The five formulated themes that guided the development of the tools and training were:

(1) Awareness about the need for palliative care in dementia;
(2) Familiarization with a person with dementia/between professionals and family caregivers;
(3) Communication about (future) care preferences as a part of advance care planning;
(4) Interprofessional collaboration during care transitions;
(5) Managing pain and responsive behavior.

## 2 Methods

### 2.1 Study design

This quantitative study used a cross-sectional design and was conducted from March 2023 to June 2025. The aims of this study were twofold. First, we describe the development of the DEDICATED questionnaire, using a stepwise approach by defining concepts, assessing the questionnaire’s face and content validity, and conducting feasibility testing, as described by de Vet et al. (2011). The second aim was to assess the questionnaire’s psychometric properties, including construct, convergent, discriminant, and concurrent validity, and internal consistency reliability. The metrics were assessed using data from DEDICATED questionnaires, which were completed by healthcare professionals who provided palliative care for people with dementia. This paper is structured into two parts to address the development and assessment of psychometric properties.

### 2.2 Development of the DEDICATED questionnaire

#### 2.2.1 Define the concepts and develop the DEDICATED questionnaire

**Fig. 1:**
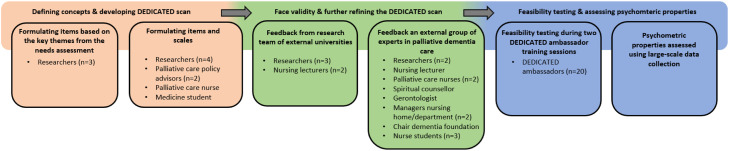
Process of developing the DEDICATED questionnaire.

Fig. 1 visualizes the steps taken to develop the DEDICATED questionnaire (De Vet et al., 2011). The qualitative and quantitative research performed within the DEDICATED project provided the theoretical basis for the concepts measured by the questionnaire (Bolt et al., 2019a; 2019b; Bolt et al., 2020, 2022; Khemai et al., 2020, 2022). Thus, the DEDICATED questionnaire contained questions related to: (1) Awareness about the need for palliative care in dementia; (2) Familiarization with a person with dementia/between professionals and family caregivers; (3) Communication about (future) care preferences as a part of advance care planning; (4) Interprofessional collaboration during care transitions; (5) Managing pain and responsive behavior. The goal of the DEDICATED questionnaire is to help healthcare professionals gain insight into their needs, learning goals, and potential areas for improvement by self-assessment of their skills and competencies related to palliative care for people with dementia. The items formulated in theme 1 (i.e., Awareness about the need for palliative care in dementia) were used to gain an impression of healthcare professionals’ perceptions of the need for palliative care in dementia. Items under themes 2, 3, 4, and 5 assessed self-perceived needs related to palliative dementia care. In March 2023, the first version of the DEDICATED questionnaire was designed by the DEDICATED research team (Fig. 1). The research team developed questions, resulting in an initial questionnaire of 22 items. In this first version, items were formulated as statements to which participants could respond using a five-point Likert scale (i.e., strongly disagree, disagree, neutral, agree, and strongly agree).

#### 2.2.2 Evaluating face validity and refining the DEDICATED questionnaire

The first version of the DEDICATED questionnaire was reviewed by experts from academia and clinical practice to evaluate the relevance, comprehensiveness, and clarity of the formulated items (De Vet et al., 2011). This established a level of face and content validity (Fig. 1). These experts included three researchers and two nursing lecturers from two collaborating research projects at other universities in the Netherlands and a separate group of 13 experts from diverse disciplines, including nursing, spiritual counselling, and gerontology. Expert feedback led to the separation of several items into multiple, more specific items, as the initial version was considered incomplete. Additionally, the items for domains 2, 3, 4, and 5 were refined to encourage critical reflection on healthcare Professional's learning goals and needs. Furthermore, a four-point Likert scale (i.e., strongly disagree, disagree, agree, and strongly agree) replaced the initial five-point version for all items. This change was recommended by the experts to stimulate healthcare professionals to reflect on their needs and learning goals by excluding the option of neutral answers. Higher sum scores of the DEDICATED questionnaire indicated higher learning needs.

#### 2.2.3 Feasibility testing

Qualtrics (i.e., an online survey system) was used as the data collection system for the DEDICATED questionnaire (Qualtrics International Inc., Provo, UT; 2024). Twenty experienced DEDICATED ambassadors were invited by mail to complete the questionnaire, focusing specifically on the content of the items (e.g., (1) phrasing of questions, (2) spelling, (3) grammar, (4) overall clarity, and (5) identification of missing aspects). In addition, we asked ambassadors to provide feedback on the usability of the questionnaire within Qualtrics (e.g., whether all answer buttons functioned properly, whether the font size was appropriate, and whether navigation through the questionnaire was clear). No concerns were identified by the ambassadors, and the DEDICATED questionnaire was considered ready for further distribution and the assessment of its psychometric properties.

### 2.3 Assessing the psychometric properties of the DEDICATED questionnaire

#### 2.3.1 Population and recruitment

We asked newly trained DEDICATED ambassadors to complete the questionnaire themselves and distribute it amongst their care team members who were: (1) direct care team members of the DEDICATED ambassador; (2) working within the field of palliative care for people with dementia; (3) in direct contact with people with dementia and their family caregivers; and (4) collaborated with other professionals during the provision of palliative care. This snowball sampling technique enabled us to collect data from a sufficient sample of healthcare professionals.

#### 2.3.2 Data collection

From June 2023, we started data collection for the psychometric evaluation of the DEDICATED questionnaire. During the first session of the DEDICATED training, DEDICATED ambassadors were asked to fill out the DEDICATED questionnaire. After the training session, ambassadors received an email containing an anonymous shareable Qualtrics link to disseminate the questionnaire to their care team members. Data from the DEDICATED ambassadors who tested the questionnaire for feasibility were also included, as no further adjustments were made to the questionnaire before further dissemination.

#### 2.3.3 Data analyses

We performed confirmatory factor analysis (CFA) and exploratory factor analysis (EFA). The CFA was conducted to analyze whether the original hypothesized factor structure of the DEDICATED questionnaire was suitable. Normality assumptions were checked before the CFA (Nussbeck et al., 2006). As these assumptions were violated, we adjusted the levels of normality using the Weighted Least Squares Mean and Variance (WLSMV) estimator (Nussbeck et al., 2006). The Comparative Fit Index (CFI), Tucker Lewis Index (TLI), Root Mean Square Error of Approximation (RMSEA), and Standardized Root Mean Square Residual (SRMR) were calculated, and interpretation was provided to indicate whether the hypothesized factor structure was suitable (Brown, 2015). Items with factor loadings of ≥.50 were retained for the CFA (Brown, 2015). The CFA was conducted using R version 4.4.2 (R Core Team, 2023) and the Lavaan package for structural equation modeling (Rosseel, 2012).

To further explore suitable factor structures, EFA was conducted using IBM SPSS version 28.0 (IBM Corp., Armonk, NY). Before starting the EFA, a test of multicollinearity was conducted for all items using the variance inflation factor (VIF) and inter-item correlations. Items with Pearson correlations of *r* ≥ .85 were removed, as these could exhibit too many overlapping aspects. The Kaiser-Meyer-Olkin (KMO) measure of sampling adequacy and Bartlett’s test for sphericity were conducted prior to factor analysis (Kaiser, 1975). An oblimin rotation method was used as we assumed that our factors would be somewhat correlated (Yong and Pearce, 2013). Factors with eigenvalues of ≥1 and items with factor loadings of ≥.40 were retained (Schreiber, 2021; Walsh et al., 2022). A second CFA was then performed using the factor structure derived from the EFA, to assess whether it provided a better fit to the data compared with the original factor structure. After the factor analyses, we calculated: (1) inter-item correlations, to assess convergent validity of the questionnaire (De Vet et al., 2011); (2) item-factor correlations for discriminant validity (Rönkkö and Cho, 2022); and (3) item-total correlations, Cronbach’s alpha (α), McDonald’s omega (ω) and a split-half test for reliability (Stensen and Lydersen, 2022). Cronbach’s α and McDonald’s ω were calculated for each factor and the complete DEDICATED questionnaire. Split-half reliability testing was assessed by randomly dividing the items of the DEDICATED questionnaire in two halves. This procedure was repeated ten times. For each division, the correlation of the sum scores of the two halves was calculated and subsequently corrected using the Spearman-Brown correction. Then, the mean of all ten corrections was calculated to obtain the mean split-half reliability coefficient (Aden et al., 2019).

To analyze whether the DEDICATED questionnaire could differentiate outcomes for different groups of healthcare professionals, we conducted an analysis of variance (ANOVA) using the Games–Howell post-hoc test to analyze scores per group of participants, as the groups may not have had equal variances (Games and Howell, 1976). For all statistically significant results (α ≤ .05), 95% confidence intervals (CI) were provided. Descriptive statistics on variables age, years of working experience, and occupation were given for the research population.

To assess concurrent validity, the DEDICATED questionnaire was administered to a second group of healthcare professionals alongside a Dutch translation of the Patient- and Family-Centered Communication (PFCC) subscale of the End-of-Life Professional Caregiver Survey (EPCS) (Lazenby et al., 2012; Ten Koppel et al., 2019). The EPCS-PFCC assesses communication skills in end-of-life care using a five-point Likert scale ranging from “not at all” (1) to “very much” (5). The EPCS was translated into Dutch for another project, in which researchers added the option “not my responsibility” to the Likert scale (Ten Koppel et al., 2019). If at least 75 % of the EPCS-PFCC was completed, any missing items or those marked “not my responsibility” were replaced with the mean of participants' completed item scores. Since lower scores on the EPCS-PFCC indicate greater needs, the scoring system was recoded to align with the DEDICATED questionnaire, where higher scores reflect higher needs. Pearson correlation coefficients were calculated between the total scores of the instruments to evaluate the DEDICATED questionnaire’s concurrent validity.

### 2.4 Ethical considerations

Ethical approval for this study was provided by the Research Ethics Commission of the Faculty of Health, Medicine, and Life Sciences at Maastricht University (Document number: FHML-REC/2023/116/Amendment2/2025_02 & FHML/HPIM/2025.986). All participants gave written informed consent for the anonymous use of the data.

## 3 Results

For the psychometric evaluation, 336 healthcare professionals returned the DEDICATED questionnaire, of which 72 % (*n* = 243) were completed. Most participants were nurses (23.9 %), and the mean age of the study population was 43.3 years (SD = 13.6). Table 1 shows the main characteristics of the population that completed the DEDICATED questionnaire.

**Table 1:** Characteristics of the population who completed the DEDICATED questionnaire.

	*n* = 243
Age, mean (SD) Years of experience in palliative care, mean (SD) Years of experience in dementia care, mean (SD) Profession, n (%)	43.3 (13.6)
	11.2 (11.4)
	14.1 (10.9)

Nurse	58 (23.9)
(Certified) nurse assistant	77 (31.7)
Elderly care physician	4 (1.6)
Spiritual counsellor	1 (0.4)
Dementia casemanager	27 (11.1)
General practitioner	1 (0.4)
Other‡	75 (30.9)
Work setting, n (%)	
Nursing home	134 (55.1)
Hospital	11 (4.5)
Home care	57 (23.5)
GP practice	0 (0.0)
Other	41 (16.9)

‡ “Other” consisted of team/ward managers, nursing students, allied care professionals, etc.

### 3.1 The DEDICATED questionnaire

The DEDICATED questionnaire was based on the five theoretical DEDICATED domains (Bolt et al., 2019a, 2019b; 2019c, 2020, 2022; Khemai et al., 2020, 2022). Domain 1 (i.e., Awareness about the need for palliative care in dementia) contained three statements concerning general knowledge about the need for palliative care. These items were not intended to assess learning needs but rather to capture healthcare professionals’ perceptions of the importance of palliative care in dementia. Therefore, items in this domain were not included in factor analyses. In addition, the DEDICATED questionnaire enquired five questions regarding the characteristics of healthcare professionals and 29 items for the needs assessment. The 29 items were scored on a four-point Likert scale (from 1 for strongly agree’’ to 4 for ‘’strongly disagree’’, with no neutral answer). The items in the developed DEDICATED questionnaire pertained to four factors, directly related to the four theoretical domains (i.e., Familiarization with a person with dementia/between professionals and family caregivers; Communication about (future) care preferences as a part of advance care planning; Interprofessional collaboration during care transitions; Managing pain and responsive behavior) with total scores ranging from 29–116 (Appendix 1). To categorize the level of needs of healthcare professionals, we conducted a K-means cluster analysis (Colledani et al., 2025). In order to provide a nuanced categorization of the needs (i.e., low, moderate, high, and very high needs), the total scores of all healthcare professionals who completed the DEDICATED questionnaire were divided into four clusters. The resulting mean cluster scores and the number of participants per cluster were used to calculate cutoff points for the questionnaire’s needs categories. Cutoff points were determined by calculating the mean of two adjacent cluster scores and subsequently rounded to the nearest integer, to match the integer-based scoring of the questionnaire (Table 2). Fig. 1 visualizes the DEDICATED questionnaire's needs categories, using the cut-off points calculated with the K-means analysis.

**Table 2:** K-means analysis for cut-off points in the DEDICATED questionnaire.

			Cluster					
			1	2		3		4
DEDICATED questionnaire mean cluster scores			50.43	70.60		85.39		105.69
Participants in cluster (n)			23	92		102		26
Cut-off points between clusters*			60.52		77.99		95.54	

*Cut-off points were determined by calculating the mean of two adjacent mean cluster scores:

Mean cluster score 1 and 2: (50.43 + 70.60) /2 = 60.52. Nearest integer = 61.

Mean cluster score 2 and 3: (70.60 + 85.39) /2 = 77.99. Nearest integer = 78.

Mean cluster score 3 and 4: (85.39 + 105.69) /2 = 95.54. Nearest integer = 96.

Fig. 2

**Fig. 2:**
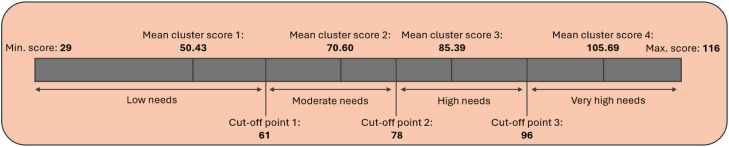
Visualization of the cut-off points for categorizing the needs in the DEDICATED questionnaire, determined by K-means analysis.

### 3.2 Psychometric properties of the DEDICATED questionnaire

The CFA revealed that the original, four-factor structure exhibited acceptable and poor fit indices (Table 3), indicating that this factor structure did not completely fit the data and that a new structure should be explored. The EFA suggested an alternative, five-factor structure. Within the EFA, VIF coefficients were between 2.242 and 4.908, indicating no severe multicollinearity between the items (Daoud, 2017). The KMO was .94 and Bartlett’s test was 5832.5 (df = 406, *p* ≤ .001), confirming that the dataset was suitable for EFA (Kaiser, 1974; Williams et al., 2010). The five factors were extracted using the eigenvalues ≥1 and Scree plot (Cattell, 1966), explaining a total variance of 71.68 % (Appendix 2). Table 4 shows the names given to factors and the possible range of scores per factor. CFA with the adjusted factors derived from the EFA showed better fit (Table 3, Appendix 3). None of the 29 items loaded onto multiple factors, meaning that the questionnaire has a good construct validity (Williams et al., 2010). Items 5 and 6 had a relatively high inter-item correlation (*r* = .80). This was also observed between items 22 and 23 (*r* = .83) and between items 28 and 29 (*r* = .79). This suggests that, within their respective factor, the items had some overlapping aspects (De Vet et al., 2011). Inter-factor correlations were between .57 and .71, indicating that the factors are correlated but measure distinct concepts (Rönkkö and Cho, 2022; Souza et al., 2017). The mean Cronbach’s α for the complete DEDICATED questionnaire was .89, and the mean McDonald’s ω was .90 (Appendix 2), indicating high internal consistency reliability (Stensen and Lydersen, 2022; Taber, 2018). All item-total correlations were above the threshold of .30, supporting the overall reliability and validity of the DEDICATED questionnaire (Ebrahimi et al., 2013). Appendix 2 and 3 show the items of the DEDICATED questionnaire, factor loadings, correlation coefficients, Cronbach’s α, and McDonald’s ω. Spearman-Brown correction of the ten random divisions showed a mean split-half coefficient of .95, further indicating strong internal consistency reliability (Aden et al., 2019).

**Table 3:** CFA fit indices.

	DEDICATED questionnaire original factor structure (four factors)		DEDICATED questionnaire adjusted factor structure (five factors)	
Fit indices*	Value	Interpretation	Value	Interpretation
CFI	.949	Acceptable fit	.961	Excellent fit
TLI	.944	Acceptable fit	.957	Excellent fit
RMSEA	.099	Poor fit	.088	Acceptable fit
SRMR	.060	Acceptable fit	.050	Excellent fit

*Comparative Fit Index (CFI), Tucker Lewis Index (TLI), Root Mean Square Error of Approximation (RMSEA), and Standardized Root Mean Square Residual (SRMR).

**Table 4:** Factors and respective labels of the DEDICATED questionnaire with minimum and maximum scores.

Factor	Label	Number of items	Item scoring
1	Familiarization with a person with dementia/between professionals and family caregivers	7	7 ± 28
2	Finding the right timing to conduct advance care planning	3	3 ± 12
3	The role of the healthcare professional in advance care planning	6	6 ± 24
4	Interprofessional collaboration during care transitions	8	8 ± 32
5	Managing pain and responsive behavior	5	5 ± 20
	Total max. score: 116		

### 3.3 Discriminative ability of the DEDICATED questionnaire

ANOVA was used to explore whether the DEDICATED questionnaire could effectively differentiate the needs and learning goals of individuals in various occupations involved in palliative care for people with dementia, with higher factor scores indicating greater needs in specific areas (Table 5). As participant numbers in the occupational categories of ‘Spiritual counsellor’ (*n* = 1), ‘General practitioner’ (*n* = 1), and ‘Elderly care physician’ (*n* = 4) were low, these data were aggregated into the occupational category ‘Other’. Mean scores of (certified) nurse assistants in Factor 3 were higher compared to the scores of nurses (mean difference = 1.82, 95% CI: .30 to 3.31, *p* = .011) and the group other occupations (mean difference = 2.11, 95 % CI: .72 to 3.49, *p* < .001). This indicates that this group exhibited greater learning needs regarding their role in initiating advance care planningand fostering collaboration with other healthcare professionals during advance care planning. Certified nurse assistants had the highest sum score on the DEDICATED questionnaire compared to the other occupational groups. They scored significantly higher statistically compared to nurses (mean difference = 6.15, 95% CI: .15 to 12.2, *p* = .042). This may suggest that the DEDICATED questionnaire can distinguish between outcomes among different groups of healthcare professionals, demonstrating a degree of discriminative ability. Other scores were not found to be statistically different. Overall, the average total score of the DEDICATED questionnaire revealed moderate and high learning needs across all occupational groups. Despite using negatively formulated questions to assess healthcare professionals’ needs, no ceiling effects were detected. This suggests that the DEDICATED questionnaire can capture the learning needs of healthcare professionals without leading them to perceive this self-assessment as exclusively negative (Baumgardner et al., 2024).

**Table 5:** DEDICATED questionnaire factor scores and total scores.

Factor labels						

	1. Familiarization with a person with dementia/between professionals and family caregivers	2. Finding the right timing to conduct ACP	3. The role of healthcare professionals in ACP	4. Inter-professional collaboration during care transitions	5. Managing pain and responsive behavior	Sum score DEDICATED questionnaire*
Occupation	Factor scoring, M (SD)					
Nurse Dementia casemanager Certified nurse assistant Other	19.3 (3.7)	8.4 (1.5)	**15.7 (3.7)**	20.6 (5.4)	12.9 (2.9)	**76.9 (14.2)**
	18.0 (4.7)	7.9 (2.0)	15.2 (4.7)	20.6 (4.6)	12.7 (3.5)	74.5 (16.0)
	20.1 (3.6)	8.9 (1.3)	**17.5 (2.7)**	22.6 (4.4)	13.9 (2.6)	**83.0 (11.9)**
	19.4 (4.4)	8.4 (2.0)	**15.4 (3.9)**	21.4 (5.8)	13.1 (3.2)	77.1 (16.9)

*Categorization of the needs: 29–61 = low needs; 62–78 = moderate needs; 79–96 = high needs; 97–116 = very high needs.

**Bold:** Statistically significant differences in scores between groups (*p* = ≤.05).

ACP - advance care planning

### 3.4 Concurrent validity of the DEDICATED questionnaire

The second examined group consisted of 146 healthcare professionals working in the field of palliative care, of whom 60% (*n* = 89) completed both the DEDICATED questionnaire and the EPCS-PFCC. A moderate positive correlation was found between the sum scores of the DEDICATED questionnaire and the EPCS-PFCC (*r* = .33, 95% CI: .13 to .50, *p* = .002) (Overholser and Sowinski, 2008). This correlation suggests that the DEDICATED questionnaire somewhat aligns with an established, validated instrument that similarly measures the needs of healthcare professionals in providing palliative care for people with dementia.

## 4 Discussion

This study aimed to develop and evaluate the psychometric properties of the DEDICATED questionnaire, a self-assessment tool for measuring healthcare professionals’ skill development needs. The DEDICATED questionnaire showed good construct validity, including convergent and discriminant validity, and internal consistency. However, some psychometric properties of the DEDICATED questionnaire require further elaboration. Specifically, six items of the questionnaire showed relatively high inter-item correlations within their respective factors. This may indicate that these questions contain overlapping content and could be redundant. However, there is currently no consensus on a threshold that indicates an inter-item correlation is too high. De Vet et al. (2011) states that inter-item correlations of ≥ .70 indicate overlapping aspects, while Paulsen & BrckaLorenz (2017) argue that values can range from .15 to .85. Meanwhile, it is important to consider the underlying theories and literature used to develop the items of a questionnaire. This can provide a rationale for retaining certain items, even when their correlation coefficients indicate a degree of overlap.

Within the DEDICATED questionnaire, item 5 addresses the identification of what an individual finds meaningful in life, whereas item 6 focuses on recognizing preferences, such as hobbies, music, physical activities, and food choices, which also impact quality of life (Jones et al., 2020). These items were kept separate, as healthcare professionals may struggle to develop a comprehensive understanding of a persons' broader wishes and values. In practice, the identification of treatment preferences often takes priority due to time constraints, risking the neglect of other meaningful aspects for the person with dementia (Midtbust et al., 2018; Ostaszkiewicz et al., 2018). However, palliative care emphasizes the importance of adopting a holistic perspective that extends beyond illness-related care needs and values (Van der Steen et al., 2014). Similarly, Bolt et al. (2022) highlighted the importance of recognizing the person behind the disease and their valued activities. The DEDICATED questionnaire retains these items separately to raise awareness and help healthcare professionals reflect on their learning needs in understanding people with dementia holistically. Items 25 and 26 also showed similarities. Item 25 enquired about healthcare professionals’ awareness of their own role in the process of transitioning from home to a nursing home, while item 26 enquired about healthcare professionals’ ability to identify co-workers who have a role in this transition. Keeping these items separated is supported by Khemai et al. (2020); in their study, healthcare professionals expressed the need to identify and approach colleagues for interprofessional collaboration and to clarify their own roles during the transition of individuals with dementia to a nursing home. Items 28 and 29 both concern pain management for people with dementia, but assess fundamentally different aspects: family involvement and the involvement of co-workers, respectively. These are both essential parts of palliative care provision for people with dementia (Fry et al., 2017). However, family caregivers are often excluded from pain management decisions, even though their knowledge of the affected persons' history means they can have valuable contributions regarding dealing with pain and responsive behavior (Fry et al., 2017). The literature review by Pu et al. (2023) also highlighted healthcare professionals’ need to involve other healthcare professionals and share knowledge in pain management for people with dementia. Therefore, both items were retained.

The occupational groups in this study indicated a moderate or high overall need for improving their skills and competencies with regard to palliative care for people with dementia. Particularly, conducting advance care planningconversations with people with dementia and family caregivers was found to be an important aspect for improvement. Conducting advance care planningwith people with dementia remains challenging (Bolt et al., 2019a; Kerr et al., 2020; Wendrich-van Dael et al., 2020), and several aspects may explain the higher needs indicated by healthcare professionals in the DEDICATED questionnaire. For example, healthcare professionals may feel uncertain about treatment decisions when advance care planningis not conducted in a timely manner, care recipients are not directly involved in advance care planning, or treatment preferences change over time (Brazil et al., 2018). An underlying challenge in proactive advance care planningdiscussions is determining the right time to initiate such conversations (Kerr et al., 2020). Timely initiation is especially important for people with dementia, as cognitive decline can hinder communication about their wishes and palliative care needs (Harrison Dening et al., 2019). However, individuals vary in their perceptions of when and how to begin discussing end-of-life and palliative care (van der Steen et al., 2016). While some may fear discussing these topics due to uncertainty about the future or loss of control (Sellars et al., 2019), others find that such discussions bring clarity and peace (de Boer et al., 2012). The uncertainty surrounding the timing of advance care planningand how healthcare professionals can effectively support these conversations may explain the higher scores on this topic in the DEDICATED questionnaire (de Boer et al., 2012, 2024; van der Steen et al., 2016). Additional fundamental aspects of communication regarding the wishes and needs for palliative care include active listening, non-verbal communication, and building trust (Peerboom et al., 2023). However, applying these skills can be challenging for healthcare professionals, as these elements are highly personal and can depend on readiness and emotions of people with dementia and relatives. Although the DEDICATED questionnaire does not directly ask professionals to self-assess these aspects, they are indirectly addressed in factor 1 (i.e., Familiarization with a person with dementia/between professionals and family caregivers) in which healthcare professionals get to know the person with dementia through communication skills and building a trustful relationship.

The DEDICATED questionnaire detected several statistically significant differences in sum scores between the nurses and (certified) nurse assistants, indicating that nurse assistants experience a greater need to work on their skills and competencies related to palliative care for people with dementia. A study by Karacsony et al. (2018) also indicated that nursing assistants felt a greater need to improve their skills and competencies for palliative care compared to nurses, as they had received less training. In addition, nurse assistants may face greater challenges in specific domains, such as providing emotional support for and communication with people with dementia and family caregivers (Karacsony et al., 2018). This could explain the higher scores in this specific topic of the DEDICATED questionnaire. On the other hand, several factors could influence the self-assessment of nurse assistants and other occupations. For instance, in a paper by White et al. (2021), nurse assistants assessed themselves as having more general knowledge about palliative care compared to nurses, as they have more frequent contact with people with dementia and can therefore draw knowledge from their experiences in the field. This is contradictory to our results, as nurse assistants indicated higher needs. Scientific literature confirms that aspects such as work environment, workload, and education can influence one’s self-assessment (Tsutsumi and Sekido, 2015). Moreover, it is important to consider that, when healthcare professionals lack awareness of their own knowledge gaps, it may reflect a state of “unconscious (in)competence” (Lane and Roberts, 2022) and could explain different needs between occupational groups.

### 4.1 Limitations

Several limitations must be considered when interpreting the results. Firstly, a snowball sampling technique was used to collect data from DEDICATED ambassadors and their involved team members. To ensure confidentiality of the DEDICATED ambassadors and other involved team members, we distributed the DEDICATED questionnaire by e-mail using anonymous invitation links. These links were shared with DEDICATED ambassadors, who subsequently disseminated them to their team members, allowing us to collect a large and diverse dataset. However, the use of anonymous links prevented us from linking responses to specific individuals, making it impossible to identify the same participants to complete the questionnaire for test-retest reliability purposes. Although test-retest reliability could not be evaluated in this study, the DEDICATED questionnaire demonstrated good internal consistency and high item-total correlations. Furthermore, factor analysis indicated that each item loaded onto a single factor, supporting the psychometric quality of the questionnaire. A second limitation of the use of a snowball sampling technique is the possibility of selection bias. DEDICATED ambassadors were asked to distribute the questionnaire to their direct team members, allowing inclusion of a broader group of healthcare professionals involved in palliative dementia care. However, it may have occurred that only the most motivated or critical team members were approached to complete the questionnaire, possibly resulting in a distorted image of the needs assessment. In addition, the use of a snowball sampling technique resulted in an imbalance regarding the occupational groups involved in this study. For instance, the number of spiritual counsellors and physicians in this study was disproportionately low compared to nursing staff. Nursing staff were often selected for the DEDICATED training and implementation, as they provide the most (palliative) care and are in close contact with people with dementia and their family caregivers. Moreover, physicians and spiritual counsellors were involved in fewer numbers and thus less frequently represented in the training and questionnaire, explaining the imbalance in our sample. Consequently, conducting comparative analyses for these groups was challenging. Thirdly, the high inter-item correlation between several items of the questionnaire could indicate redundancy. Even though scientific literature provides a theoretical justification to keep the items separate, future research will need to comprise sensitivity analyses of the questionnaire’s factor structure to analyze how the questionnaire functions when items with high inter-item correlations are merged, deleted or rephrased. Lastly, the DEDICATED questionnaire is currently used in the Dutch palliative care context. Cultural and linguistic factors, such as communication norms, attitudes toward palliative care, and healthcare system structures, may influence how questions are understood and answered in other settings. This affects the generalizability of the DEDICATED questionnaire. As DEDICATED is currently implemented in the Dutch Caribbean, where linguistic and cultural differences are present, further application and validation efforts will be conducted. For instance, healthcare professionals will be enquired to critically reflect whether the questions of the DEDICATED questionnaire apply to palliative care activities in their specific context. This ensures that a culturally-adapted version of the questionnaire is applied and validated. Similarly, the English version of the DEDICATED questionnaire is currently not validated and will require substantial data collection from English-speaking healthcare professionals.

## 5 Conclusion

The DEDICATED questionnaire demonstrates promising psychometric properties and has the potential to identify the diverse needs of various healthcare professionals in palliative dementia care. Healthcare professionals can use the DEDICATED questionnaire to gain insights into their learning needs. As the DEDICATED project expands, the questionnaire will be administered to a larger population of healthcare professionals. This could be a starting point for knowledge acquisition and skills improvement, further improving the quality of palliative care provided to people with dementia and their family caregivers.

## Data availability

The data that support the findings of this study are available from the corresponding author upon reasonable request.

## CRediT authorship contribution statement

**Jesper M.A. Biesmans:** Writing – original draft, Visualization, Validation, Methodology, Investigation, Formal analysis, Data curation, Conceptualization. **Sascha R. Bolt:** Writing – review & editing, Writing – original draft, Supervision, Conceptualization. **Sandra M.G. Zwakhalen:** Writing – review & editing, Supervision, Methodology. **Daisy J.A. Janssen:** Writing – review & editing, Writing – original draft, Supervision. **Judith M.M. Meijers:** Writing – review & editing, Writing – original draft, Supervision, Methodology, Conceptualization.

## Declaration of competing interest

The authors declare the following financial interests/personal relationships which may be considered as potential competing interests:

Jesper M.A. Biesmans reports financial support was provided by The Netherlands Organization for Health Research and Development (ZonMw). If there are other authors, they declare that they have no known competing financial interests or personal relationships that could have appeared to influence the work reported in this paper.

## Appendix Supplementary materials

Image, application 1

Image, application 2

Image, application 3
