# Supplementary material for: Development and psychometric evaluation of a learning needs assessment tool for healthcare professionals in palliative dementia care: A cross-sectional study
Source: Int J Nurs Stud Adv. 2025 Nov 14;9:100455. doi: 10.1016/j.ijnsa.2025.100455 (PMC12670527; doi:10.1016/j.ijnsa.2025.100455)
Supplement: Supplementary file 1 [file mmc1.docx]

Z

**DEDICATED** Scan

www.dedicatedwerkwijze.nl


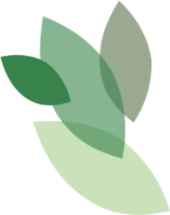
Z

**DEDICATED is about palliative care for people with dementia. This DEDICATED Scan helps healthcare professionals to identify their needs and learning goals, by reflecting on their skills and competencies. This insight supports the team in deciding which DEDICATED tools to start working with first. The statements below concern palliative care for people with dementia and their loved ones. For each statement, circle the answer that best applies to you.**

**1. The DEDICATED-training:**

- I attended the First DEDICATED-training
- I attended the Second DEDICATED-training
- I completed the DEDICATED-training
- I did not follow the DEDICATED-training

**Subscore:**

**Please indicate to what extent you agree with the following statements: My score:**

1. I know what palliative care for people with dementia entails. 1 2 3 4

2. Palliative care is applicable from the time of diagnosis. 1 2 3 4

3. I feel competent in providing palliative care to people with dementia. 1 2 3 4

Strongly disagree

Disagree

Agree

Strongly agree

**①**

**④**

**②**

**③**

**Awareness about the need for palliative care**

**4. I work as a:**

- (Home care) nurse
- Dementia case manager
- General Practitioner
- Psychologist
- Spiritual counsellor
- Elderly care physician
- Nurse assistant
- Other, namely: ………………….

**3. I work at a:**

- Nursing home
- Hospital
- GP-practice
- Home care organization
- Other, namely: ……………………

**7. How many years of working experience
do you have in care for people with dementia?**

(Please fill out in full years): ………………….

**6. How many years of working experience
do you have in palliative care?**

(Please fill out in full years): ………………….

**5. What is your age?**

(Please fill out in full years): ……………………

**2. Date of the DEDICATED-training:**

- - Date (dd/mm/yy): ………………….
  - Not applicable

**Characteristics**

**DEDICATED** Scan

www.dedicatedwerkwijze.nl

Strongly disagree

Disagree

Agree

Strongly agree

**①**

**④**

**②**

**③**

1. **Familiarization with a person with dementia/between professionals and family caregivers**

**Subscore:**

**2. Finding the right timing to conduct advance care planning**

**Please indicate to what extent you agree with the following statements: My score:**

***I need more tools/support to:***

8. Timely discuss wishes and possibilities for future care with the person with dementia. 1 2 3 4

9. Timely involve the relatives in these discussions. 1 2 3 4

10. Determine when the person and relatives are ready for these discussions. 1 2 3 4

**Subscore:**

**3. The role of the healthcare professional in advance care planning**

**Please indicate to what extent you agree with the following statements: My score:**

***I need more tools/support to:***

11. Determine when I as a care professional, am ready for these discussions. 1 2 3 4

12. Determine when follow-up discussions can be conducted. 1 2 3 4

13. Know what my role is in conducting these discussions. 1 2 3 4

14. Know where I can document the decisions made in these discussions. 1 2 3 4

15. Collaborate with other disciplines around advance care planning. 1 2 3 4

16. Collaborate with other organizations around advance care planning. 1 2 3 4

**Subscore:**

**Please indicate to what extent you agree with the following statements: My score:**

***I need more tools/support to:***

1. Get to know the biography of a person with dementia. 1 2 3 4

2. Get to know the personal characteristics. 1 2 3 4

3. Get to know the relatives (family and loved ones). 1 2 3 4

4. Get to know the content of the care plan or dossier. 1 2 3 4

5. Recognize what is important to someone. 1 2 3 4

6. Recognize what someone likes to do. 1 2 3 4

7. Recognize what the wishes for future care are. 1 2 3 4


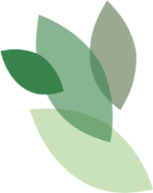

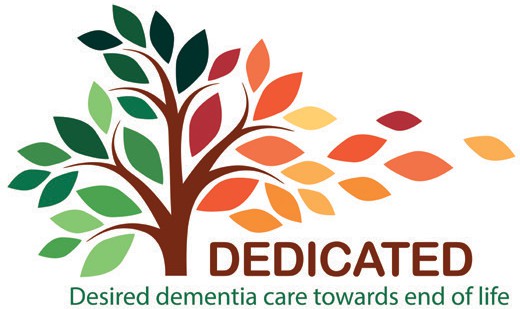


Strongly disagree

Disagree

Agree

Strongly agree

**①**

**④**

**②**

**③**

**4. Interprofessional collaboration during care transitions**

**5. Managing pain and responsive behaviour**

**Please indicate to what extent you agree with the following statements: My score:**

***I need more tools/support to:***

25. Assess whether someone is in pain. 1 2 3 4

26. Know what to do if I notice someone is in pain. 1 2 3 4

27. Deal with responsive behaviour. 1 2 3 4

28. Collaborate with my colleagues in cases of pain and responsive behavior. 1 2 3 4

29. Collaborate with the relatives in cases of pain and responsive behavior. 1 2 3 4

**Subscore:**

**Total score DEDICATED Scan:**

**Subscore:**

**Please indicate to what extent you agree with the following statements: My score:**

***I need more tools/support to:***

17. Prepare myself in a timely manner for relocation of someone with dementia. 1 2 3 4

18. Prepare the person with dementia in a timely manner for a relocation. 1 2 3 4

19. Prepare the relatives in a timely manner for a relocation. 1 2 3 4

20. Ensure a warm farewell / welcome with attention for the person with dementia. 1 2 3 4

21. Transfer information to the receiving organization during a relocation. 1 2 3 4

22. Know what my own role as a caregiver is during a relocation. 1 2 3 4

23. Know who is responsible for each task during a relocation. 1 2 3 4

24. Enhance cooperation with other involved parties during a relocation. 1 2 3 4

www.dedicatedwerkwijze.nl
