# Supplementary material for: Development and psychometric evaluation of a learning needs assessment tool for healthcare professionals in palliative dementia care: A cross-sectional study
Source: Int J Nurs Stud Adv. 2025 Nov 14;9:100455. doi: 10.1016/j.ijnsa.2025.100455 (PMC12670527; doi:10.1016/j.ijnsa.2025.100455)
Supplement: Supplementary file 2 [file mmc2.docx]

| Appendix 1. Exploratory factor analysis of the DEDICATED scan. | | | | | | | | | | |  |
| --- | --- | --- | --- | --- | --- | --- | --- | --- | --- | --- | --- |
|  | Items | Factors | | | | | Inter-item  Correlations M (±) | Item-total  correlations | Cronbach’s  α | | McDonald’s  ω |
|  | ***I need more tools/support to:*** | 1 | 2 | 3 | 4 | 5 |  |  | |  |  |
| 1 | Get to know the biography of a person with dementia. | .716 |  |  |  |  | .647 (.541 ± .800) | .767 | | .927 | 0.922 |
| 2 | Get to know the personal characteristics. | .856 |  |  |  |  |  | .814 | |  |  |
| 3 | Get to know the relatives (family and loved ones). | .765 |  |  |  |  |  | .729 | |  |  |
| 4 | Get to know the content of the care plan or dossier. | .629 |  |  |  |  |  | .725 | |  |  |
| 5 | Recognize what is important to someone. | .823 |  |  |  |  |  | .821 | |  |  |
| 6 | Recognize what someone likes to do. | .880 |  |  |  |  |  | .824 | |  |  |
| 7 | Recognize what the wishes for future care are. | .678 |  |  |  |  |  | .718 | |  |  |
| 8 | Timely discuss the wishes and possibilities for future care with the person  with dementia. |  | .409 |  |  |  | .616 (.475 ± .724) | .664 | | .829 | 0.836 |
| 9 | Timely involve the relatives in these discussions. |  | .463 |  |  |  |  | .799 | |  |  |
| 10 | Determine when the person and relatives are ready for these discussions. |  | .568 |  |  |  |  | .509 | |  |  |
| 11 | Determine when I myself as a care professional, am ready for these discussions. |  |  | .542 |  |  | .619 (.492 ±.769) | .706 | | .907 | 0.915 |
| 12 | Determine when follow-up discussions can be conducted. |  |  | .509 |  |  |  | .718 | |  |  |
| 13 | Know what my role is in conducting these discussions. |  |  | .576 |  |  |  | .757 | |  |  |
| 14 | Know where I can document the decisions made in these discussions. |  |  | .766 |  |  |  | .796 | |  |  |
| 15 | Collaborate with other disciplines around advance care planning. |  |  | .722 |  |  |  | .756 | |  |  |
| 16 | Collaborate with other organizations around advance care planning. |  |  | .731 |  |  |  | .727 | |  |  |
| 17 | Prepare myself in a timely manner for relocation of someone with dementia. |  |  |  | .622 |  | .671 (.511 ± .833) | .727 | | .942 | 0.943 |
| 18 | Prepare the person with dementia in a timely manner for a relocation. |  |  |  | .753 |  |  | .764 | |  |  |
| 19 | Prepare the relatives in a timely manner for a relocation. |  |  |  | .745 |  |  | .814 | |  |  |
| 20 | Ensure a warm farewell and/or welcome with attention for the person with dementia. |  |  |  | .585 |  |  | .710 | |  |  |
| 21 | Transfer information to the receiving organization during a relocation. |  |  |  | .815 |  |  | .844 | |  |  |
| 22 | Know what my own role as a caregiver is during a relocation. |  |  |  | .807 |  |  | .831 | |  |  |
| 23 | Know who is responsible for each task during a relocation. |  |  |  | .863 |  |  | .818 | |  |  |
| 24 | Enhance cooperation with other involved parties during a relocation. |  |  |  | .783 |  |  | .833 | |  |  |
| 25 | Assess whether someone is in pain. |  |  |  |  | .784 | .630 (.498 ± .799) | .680 | | .895 | 0.900 |
| 26 | Know what to do if I notice someone is in pain. |  |  |  |  | .740 |  | .748 | |  |  |
| 27 | Deal with responsive behaviour. |  |  |  |  | .701 |  | .736 | |  |  |
| 28 | Collaborate with my colleagues in cases of pain and responsive behavior. |  |  |  |  | .783 |  | .781 | |  |  |
| 29 | Collaborate with the relatives in cases of pain and responsive behavior. |  |  |  |  | .648 |  | .766 | |  |  |
