# Supplementary material for: Development and psychometric evaluation of a learning needs assessment tool for healthcare professionals in palliative dementia care: A cross-sectional study
Source: Int J Nurs Stud Adv. 2025 Nov 14;9:100455. doi: 10.1016/j.ijnsa.2025.100455 (PMC12670527; doi:10.1016/j.ijnsa.2025.100455)
Supplement: Supplementary file 3 [file mmc3.docx]

| Appendix 2. Confirmatory factor analysis of unadjusted and adjusted structure of the DEDICATED scan | | | | | | | | | | |
| --- | --- | --- | --- | --- | --- | --- | --- | --- | --- | --- |
|  | Items | Factors (original structure) | | | | Factors (adjusted structure) | | | | |
|  | ***I need more tools/support to:*** | 1 | 2 | 3 | 4 | 1 | 2 | 3 | 4 | 5 |
| 1 | Get to know the biography of a person with dementia. | .812 |  |  |  | .868 |  |  |  |  |
| 2 | Get to know the personal characteristics. | .794 |  |  |  | .894 |  |  |  |  |
| 3 | Get to know the relatives (family and loved ones). | .705 |  |  |  | .785 |  |  |  |  |
| 4 | Get to know the content of the care plan or dossier. | .842 |  |  |  | .871 |  |  |  |  |
| 5 | Recognize what is important to someone. | .817 |  |  |  | .912 |  |  |  |  |
| 6 | Recognize what someone likes to do. | .815 |  |  |  | .903 |  |  |  |  |
| 7 | Recognize what the wishes for future care are. | .754 |  |  |  | .849 |  |  |  |  |
| 8 | Timely discuss the wishes and possibilities for future care with the person with dementia. |  | .708 |  |  |  | .852 |  |  |  |
| 9 | Timely involve the relatives in these discussions. |  | .828 |  |  |  | .974 |  |  |  |
| 10 | Determine when the person and relatives are ready for these discussions. |  | .629 |  |  |  | .774 |  |  |  |
| 11 | Determine when I myself as a care professional, am ready for these discussions. |  | .694 |  |  |  |  | .808 |  |  |
| 12 | Determine when follow-up discussions can be conducted. |  | .747 |  |  |  |  | .869 |  |  |
| 13 | Know what my role is in conducting these discussions. |  | .780 |  |  |  |  | .891 |  |  |
| 14 | Know where I can document the decisions made in these discussions. |  | .761 |  |  |  |  | .874 |  |  |
| 15 | Collaborate with other disciplines around advance care planning. |  | .769 |  |  |  |  | .921 |  |  |
| 16 | Collaborate with other organizations around advance care planning. |  | .707 |  |  |  |  | .863 |  |  |
| 17 | Prepare myself in a timely manner for relocation of someone with dementia. |  |  | .767 |  |  |  |  | .805 |  |
| 18 | Prepare the person with dementia in a timely manner for a relocation. |  |  | .770 |  |  |  |  | .849 |  |
| 19 | Prepare the relatives in a timely manner for a relocation. |  |  | .863 |  |  |  |  | .920 |  |
| 20 | Ensure a warm farewell and/or welcome with attention for the person with dementia. |  |  | .768 |  |  |  |  | .835 |  |
| 21 | Transfer information to the receiving organization during a relocation. |  |  | .868 |  |  |  |  | .908 |  |
| 22 | Know what my own role as a caregiver is during a relocation. |  |  | .850 |  |  |  |  | .927 |  |
| 23 | Know who is responsible for what during a relocation. |  |  | .819 |  |  |  |  | .919 |  |
| 24 | Enhance cooperation with other involved parties during a relocation. |  |  | .846 |  |  |  |  | .920 |  |
| 25 | Assess whether someone is in pain. |  |  |  | .680 |  |  |  |  | .774 |
| 26 | Know what to do if I notice someone is in pain. |  |  |  | .817 |  |  |  |  | .881 |
| 27 | Deal with responsive behaviour. |  |  |  | .807 |  |  |  |  | .877 |
| 28 | Collaborate with my colleagues in cases of pain and responsive behavior. |  |  |  | .804 |  |  |  |  | .893 |
| 29 | Collaborate with the relatives in cases of pain and responsive behavior. |  |  |  | .861 |  |  |  |  | .950 |
